# Association between surgical technique, adhesions and morbidity in women with repeat caesarean section: a retrospective study in a rural hospital in Western Tanzania

**DOI:** 10.1186/s12884-020-03229-8

**Published:** 2020-10-04

**Authors:** R. Mooij, I. H. Mwampagatwa, J. van Dillen, J. Stekelenburg

**Affiliations:** 1 Ndala Hospital, 15, Ndala, Tanzania; 2 Jeroen Bosch Hospital, Henri Dunantstraat 1, 5223 GZ ‘s-Hertogenbosch, The Netherlands; 3 College of Health Sciences, University of Dodoma, 395, Dodoma, Tanzania; 4 Radboud University Medical Centre, Geert Grooteplein-Zuid 10, 6525 GA Nijmegen, The Netherlands; 5 Leeuwarden Medical Centre, Henri Dunantweg 2, 8934 AD Leeuwarden, The Netherlands; 6 University Medical Centre Groningen/University of Groningen, Antonius Deusinglaan 1, 9700 AD Groningen, The Netherlands

**Keywords:** Caesarean section, Tissue adhesions, Outcome, Trial of labour

## Abstract

**Background:**

The worldwide incidence of birth by Caesarean Section (CS) is rising. Many births after a previous CS are by repeat surgery, either by an elective CS or after a failed trial of labour. Adhesion formation is associated with increased maternal morbidity in patients with repeat CSs. In spite of large-scale studies the relation between the incidence of adhesion formation and CS surgical technique is unclear. This study aims to assess maternal and neonatal morbidity and mortality after repeat CSs in a rural hospital in a low-income country (LIC) and to analyse the effect of surgical technique on the formation of adhesions.

**Methods:**

A cross-sectional, retrospective medical records study of all women undergoing CS in Ndala Hospital in 2011 and 2012.

**Results:**

Of the 3966 births, 450 were by CS (11.3%), of which 321 were 1^st^ CS, 80 2^nd^ CS, 36 3^rd^ CS, 12 4^th^ and one 5^th^ CS (71, 18, 8, 3 and 0.2% respectively). Adhesions were considered to be severe in 56% of second CSs and 64% of third CSs. In 2^nd^ CSs, adhesions were not associated with closure of the peritoneum at 1^st^ CS, but were associated with the prior use of a midline skin incision. There was no increase in maternal morbidity when severe adhesions were present. Adverse neonatal outcome was more prevalent when severe adhesions were present, but this was statistically non-significant (16% vs 6%).

**Conclusions:**

Our results give insight into the practice of repeat CS in our rural hospital. Adhesions after CSs are common and occur more frequently after midline skin incision at 1^st^ CS compared to a transverse incision. Reviewing local data is important to evaluate quality of care and to compare local outcomes to the literature.

## Background

Caesarean section is the most commonly performed abdominal surgical procedure, and the incidence is rising worldwide [1, 2]. The percentage of births by Caesarean Section (CS) is low in the eastern and southern African region (6.2%), but there is a large variation between and within African countries [1, 3]. In the case of previous CS, sometimes CS is the only option offered, but in most settings, women can either choose an elective CS or a trial of labour (TOL) [4]. In low-income countries (LICs), both the risks of TOL as well as the risks of repeat CS are increased compared to high-income countries [5–8]. Maternal mortality after CS in Africa is 50 times higher than in high-income countries (HICs), mostly due to anaesthesia complications and haemorrhage [9, 10]. The most feared complication of TOL is uterine rupture (UR), which occurs in 0.47% of women who have a TOL [11]. In low-income countries, UR is reported to occur in up to 6.7% of cases, but these numbers are not reliable since these studies describe only in-hospital deliveries and do not report population data [12, 13]. Adhesion formation after surgery can be the cause of chronic pain and infertility; both common long-term maternal morbidities after CS, as well as a reason for readmission and repeated surgery [14]. Adhesions are associated with both a longer operation time and intra-operative morbidity such as bladder lesions [14, 15]. In subsequent pregnancies, the advantages of vaginal birth after CS (VBAC) are more relevant, since the risks of UR and abnormal placentation increase with the number of previous CS [16]. In many African countries, the total fertility rate (TFR) is high, making VBAC after previous CS an essential strategy in reducing the rising rate of CSs and its associated morbidities [17]. Rates of VBAC vary between 38 and 48% across African countries and hospitals [13, 17, 18].

Internationally, there is debate concerning surgical CS techniques and adhesion formation. Two large meta-analyses examined closure of the visceral peritoneum and reported opposite conclusions [19, 20]. In Ndala Hospital, Tabora Region, Tanzania, different surgical techniques for CS are in use, which are closure and non-closure of the visceral peritoneum. We performed a retrospective study to assess maternal and neonatal morbidity and mortality after repeat CS in this rural hospital in an LIC and evaluated the effect of surgical technique on the formation of adhesions.

## Methods

### Setting

This study was done at Ndala Hospital, a faith-based hospital, situated in a rural part of Western Tanzania in the Tabora region. The prevalence of CS in this region, as reported in the Tanzania demographic and health survey, was 2.7% in 2015–2016 [21, 22]. In the Tabora region, 54% of deliveries were assisted by a skilled birth attendant (country average: 64%), and the regional TFR was 6.7 (country average: 5.2) [22].

The hospital serves a catchment area of approximately 200,000 people. Annually, there are roughly 2200 births in the hospital. There is no maternity waiting home. Both basic and comprehensive emergency obstetric care are available. The foetal condition is monitored by intermittent auscultation. Standardised operative reports for CS were introduced in 2010 and include the indication for surgery, previous CS (and number) and a brief section about the presence of adhesions. These were added to the patient’s record after surgery.

Most women with one previous CS, without an indication for a CS or contra-indication for VBAC, choose TOL. Internationally, most contra-indications for TOL are relative and not absolute [23]. Contra-indications for TOL in Ndala Hospital consisted of a non-cephalic presentation, and an elective CS recommended by the surgeon at the time of the previous CS (for example due to a uterine rupture or a classical incision, Table 2). In women with more than one previous CS, elective repeat CS is performed, unless the woman requests TOL or presents in the 2^nd^ stage of labour.

### Participants

All women undergoing CS in Ndala Hospital between March 2011 and December 2012 were analysed. Patients with a CS were identified using the operating theatre logbook. The delivery logbook was used to obtain information on neonatal outcomes. To assess the relationship between surgical technique and adhesions, for all 2^nd^ and 3^rd^ CSs, patient records and operative reports were analysed for demographics, indication for CS, whether bilateral tubal ligation was done, and maternal and neonatal outcome. Presence and severity of adhesions were scored in the standardised operative report and divided into “severe” or “minor or no” adhesions, according to the subjective assessment of the operating doctor. If the previous CS was conducted in Ndala and the records were complete, surgical technique (closure of which layers) during prior CS was noted. CSs were divided in elective and emergency procedures. Elective CS was defined as a CS planned before the start of labour and emergency CS was chosen when the decision was made after the onset of labour (or after TOL). Adverse perinatal outcome was defined as a low Apgar score (< 7 at 5′), including stillbirth [24].

### Statistical analysis

Data management was done using Microsoft® Office Excel® 2007; statistical analysis was done with Epi Info™ 7 (Centers for Disease Control). First and second repeat CS were compared to each other and CS with minor adhesions were compared to CS with severe adhesions. *P-* values were calculated with Fisher’s exact tests, T-tests and Kruskal-Wallis tests, as appropriate for the data type and distribution.

## Results

Among the 3966 women who gave birth in the hospital during the 22-month study period, there were 450 CSs (11.3%) of which 321 (71%) were 1^st^ CS, 80 (18%) were 2^nd^ CS, 36 (8%) were 3^rd^ CS, 12 (3%) were 4^th^, and one was a 5^th^ CS (0.2%). In 99% of patients (115/116) who underwent a 2^nd^ or 3^rd^ CS, presence and severity of adhesions were reported. Adhesions were considered to be severe in 56% (44/79) of 2^nd^ CS and in 64% (23/36) of 3^rd^ CS (*p* = 0.08). For 1^st^ CS, the skin incision was more often transverse when operated in Ndala, compared with those who underwent surgery in other hospitals: 71% (54/76) vs 8% (3/38), *p* < 0.01. The baseline characteristics of women with repeat CS (data for 2^nd^ and 3^rd^ CS only) are shown in Table 1.

**Table 1:** Characteristics of women with repeat CS

**Operative characteristics**			
**Median estimated blood loss**	150 (100–300)	225 (150–300	0.98
**(ml, interquartile range)**	(*n* = 78)	(*n* = 36)	
**Classical incision in uterus**	1 (1%)	2 (6%)	0.23
**Inadequate lower segment**	15 (19%)	10 (28%)	0.27
**Total number tube ligation**	26 (33%)	27 (75%)	0.01
**Skin incision**			
Midline	36 (45%)	25 (69%)	0.01
Transverse	43 (54%)	11 (31%)	0.02
Combined (T-incision)	1 (1%)	0	0.69
**Adhesions (total)**	***n*** **= 79**	***n*** **= 36**	
Minor	35 (44%)	23 (64%)	0.08
Severe	44 (56%)	13 (36%)	
**Neonatal outcome**	***n*** **= 82**	***n*** **= 32**	
Stillbirth	8 (10%)	0	0.05
Child alive, Apgar score < 7 at 5’	2 (2%)	0	0.48
Adverse neonatal outcome (stillbirth and Apgar score < 7 at 5)	10 (12%)	0	0.02
**Hospital previous CS**	***n*** **= 80**	***n*** **= 72**	
Ndala	57 (71%) 4	8 (67%)	0.54
Other hospital	23 (29%)	21 (29%)	0.95
Unknown	0	3 (4%)	0.10

Adverse perinatal outcomes were observed more often (12% vs 0%, *p* = 0.02) in women with 2^nd^ CS than 3^rd^ CS. Thirty-eight of 49 women (78%) who were booked for an elective repeat CS were in labour before the planned surgery. Adverse neonatal outcome was more common when severe adhesions were present (16% vs 6%), but this was not statistically significant (*p* = 0.14, Table 2). A transverse skin incision was statistically significantly associated with fewer adhesions than a midline incision.

**Table 2:** Severity of adhesions in second CS^a^

**Median age (yrs, interquartile range)**	**22 (20–26)**	**25 (20–30)**	**0.04**
**Median blood loss (ml, interquartile range)**	**150 (100–400)**	**175 (120–300)**	**0.79**
**Neonatal outcome**	***n*** **= 45**	***n*** **= 36**	
Stillbirth	6 (14%)	1 (3%)	0.10
Child alive, Apgar Score of < 7 at 5’	1 (2%)	1 (3%)	0.69
Adverse neonatal outcome (stillbirth or Apgar Score < 7 at 5)	7 (16%)	2 (6%)	0.14
**Characteristics at 1st CS**			
Uterotomy closure in 2 layers	59% (19/32)	71% (15/21)	0.55
Transverse incision	35% (15/43)	62%(21/34)	0.03
Closure visceral peritoneum	59% (19/32)	53% (16/30)	0.82
Closure rectus muscles	16%(5/32)	20% (4/20)	0.72
Wound infection	14% (3/22)	6%(1/16)	0.62

Of the 13 cases with multiple repeat CS (12 women with a 4^th^ CS, one with a 5^th^ CS), all neonates had Apgar scores above seven at 5 min, and no neonatal or severe acute maternal morbidity or mortality was recorded.

## Discussion

In this rural hospital in an LIC, severe adhesions after CS are common. Adverse neonatal outcome is observed in 12% of 2^nd^ CS, while this was not the case in 3^rd^ CS. Half of the patients had a midline incision during their first CS, which was associated with severe adhesions.

The majority of patients (78%) who were booked for an elective repeat CS were in labour before the planned surgery. This has been described in a study from Uganda as well [4]. No data were collected on the scheduled surgery date, and it is unclear if the women did not come for their surgical appointment, or if labour started beforehand. The first could be because women who prefer vaginal birth are afraid they are not allowed a TOL in the hospital and deliberately arrive late, with higher risk of adverse outcome [4, 25]. The latter could be explained by the fact that no reliable gestational age was available and the planned CS was intentionally planned late to prevent accidental iatrogenic preterm birth. The statistically lower incidence of adverse perinatal outcome in 3^rd^ CS (0% compared to 12% in 2^nd^ CS, *p* = 0.02) suggests when when they are advised not to have a TOL, women present in time.

We observed a high incidence of adverse perinatal outcome after 2^nd^ CSs (12%). This might reflect the high general perinatal mortality rate in emergency CSs in a low-resource setting, which has been reported to be up to 19% [26]. In our hospital the perinatal mortality in 1^st^ CSs is unknown. The aim of this study was to evaluate surgical techniques in CS, but our research has also provided insight in the practice of TOL and repeat CS. Because of the risk of selection bias and without information on successful VBAC rates, this study is not suited to address the risks and benefits of elective CS vs TOL. However, the finding of such a high incidence of adverse perinatal outcomes in 2^nd^ CS (after TOL) warrants further research into the practice of TOL in our hospital. A new study to assess the safety of TOL after a previous CS in Ndala Hospital has already started.

This study found a prevalence of 56% of severe adhesions after the first CS. This is similar to findings of a recent study in Ghana [27]. Adhesions are a recognised cause of maternal morbidity and a frequent finding in repeat CS [28]. The relationship between adhesions and surgical technique was only examined in women with a 2^nd^ CS. Because of the smaller number of higher order repeat CS, as well as different techniques used in previous CS, finding an association was not possible for this group.

In many studies, maternal and perinatal mortality are not significantly different in women with or without adhesions. Adhesiolysis increases the time to delivery of the child and makes the costs significantly higher [29–31]. These studies were conducted in HICs, with a low general perinatal mortality, and CS were performed by medical specialists under optimal conditions. In LICs, the consequences of adhesions could be more severe, which can explain the trend we found towards a higher neonatal mortality rate.

Many studies have been conducted to identify ways to prevent adhesions by assessing surgical techniques, as well as pharmacotherapeutic “adhesion barriers” [32, 33]. Whether closure of the peritoneum during CS affects (reduces or increases) the incidence of adhesions is still debated [19, 20]. Reviews are based on uncontrolled prospective and retrospective studies. Long-term results of the Coronis RCT reassuringly showed no difference in any outcomes related to adhesions (such as chronic pain and infertility) after closure or non-closure of the peritoneum [34]. Long-term results of the CAESAR RCT are expected as well [35]. In the meantime, arguments for non-closing are the shorter operating time and the use of less suture material. Still, if closing the peritoneum reduces adhesions, this investment could easily be worthwhile. This is why some authors have already argued for the closure of the peritoneum [36]. In our retrospective study, there was no association between the closure of the peritoneum and the presence of adhesions.

The difference in the type of incision between patients operated in Ndala Hospital and other hospitals highlights the generally low rate of transverse incisions in LICs [37]. Ndala Hospital has regularly had foreign doctors performing and teaching CS using Pfannenstiel or Misgav-Ladach techniques with a transverse incision [38]. In Tanzania, many domestically educated doctors are only trained in performing CS with subumbilical midline incisions. This explains the difference in 1^st^ CS techniques found in this study (71% transverse incisions for CSs performed in Ndala, 8% when CS was performed elsewhere). The WHO advises midline incision because it is easier when using local anaesthesia [39]. However, transverse incisions have been shown beneficial for different short-term outcomes (such as pain and wound infection) in a low-resource setting [40]. RCTs in general surgery have shown an increased risk of incisional hernias in midline incisions [41]. For this, as well as for cosmetic reasons, transverse skin incision could be the technique of choice [37]. To our knowledge, no studies have examined adhesion formation following different CS skin incisions. In our research, we found significantly fewer adhesions after a previous transverse incision.

In this retrospective study, the significantly higher maternal age at second CS in women with severe adhesions could be either a finding due to bias or a biological effect. Recently, another large study has also found an increase in adhesion formation in women ≥35 years (adjusted odds ratio 1.28, 95% CI: 1.05–1.55) [42].

### Study limitations

There could be confounding by indication, as doctors could decide for themselves whether to close the peritoneum and observation bias in recognising and noting adhesions. The presence and subjective severity of adhesions was a regular part of the operating report. We stratified the presence of adhesions in two groups, without details of the location or a more precise grading, such as has been used in prospective studies [43]. Another bias could be caused by women with many adhesions being less likely to become pregnant. Since most midline incisions were done in other hospitals, different patient and doctor characteristics could be a confounder for the increased number of adhesions after a midline incision CS. Information on perinatal outcome is limited to the Apgar score at 5 min without knowledge of the condition at discharge; there is no long-term follow up or registration of perinatal mortality. Strengths of this study are both the relatively high number of patients and information at the level of rural district hospitals in an LIC, which allowed us to compare local outcomes to larger studies in the literature.

## Conclusion

This retrospective study provides insight into the practice of repeat CS in a rural hospital in an LIC. Adhesions after CSs are common and occur more frequently after midline skin incision. No effect of closure of the peritoneum on adhesion formation was observed. Adverse neonatal outcomes were not statistically significantly more common when severe adhesions were present. Audit of local data is important to evaluate the quality of care and to relate local outcomes to the literature.

## Data Availability

Data are available from the author upon request.

## Abbreviations

CS: Caesarean section
HIC: High-income country
LIC: Low-income country
RCT: Randomized controlled trial
TFR: Total fertility rate
TOL: Trial of labour
UR: Uterine rupture
VBAC: Vaginal birth after caesarean section
